# Control of Adipocyte Differentiation in Different Fat Depots; Implications for Pathophysiology or Therapy

**DOI:** 10.3389/fendo.2015.00001

**Published:** 2015-01-30

**Authors:** Xiuquan Ma, Paul Lee, Donald J. Chisholm, David E. James

**Affiliations:** ^1^Cellular Systems Biology, Diabetes and Metabolism Division, Garvan Institute of Medical Research, Sydney, NSW, Australia; ^2^Charles Perkins Centre, School of Molecular Bioscience, The University of Sydney, Sydney, NSW, Australia; ^3^Clinical Diabetes, Appetite and Metabolism, Diabetes and Metabolism Division, Garvan Institute of Medical Research, Sydney, NSW, Australia; ^4^Charles Perkins Centre, School of Molecular Bioscience, School of Medicine, The University of Sydney, Sydney, NSW, Australia

**Keywords:** adipogenesis, adipocyte, subcutaneous white adipose tissue, visceral white adipose tissue, brown adipose tissue, fat depot, adipocyte differentiation, adipose tissue

## Abstract

Adipocyte differentiation and its impact on restriction or expansion of particular adipose tissue depots have physiological and pathophysiological significance in view of the different functions of these depots. Brown or “beige” fat [brown adipose tissue (BAT)] expansion can enhance thermogenesis, lipid oxidation, insulin sensitivity, and glucose tolerance; conversely expanded visceral fat [visceral white adipose tissue (VAT)] is associated with insulin resistance, low grade inflammation, dyslipidemia, and cardiometabolic risk. The largest depot, subcutaneous white fat [subcutaneous white adipose tissue (SAT)], has important beneficial characteristics including storage of lipid “out of harms way” and secretion of adipokines, especially leptin and adiponectin, with positive metabolic effects including lipid oxidation, energy utilization, enhanced insulin action, and an anti-inflammatory role. The absence of these functions in lipodystrophies leads to major metabolic disturbances. An ability to expand white adipose tissue adipocyte differentiation would seem an important defense mechanism against the detrimental effects of energy excess and limit harmful accumulation of lipid in “ectopic” sites, such as liver and muscle. Adipocyte differentiation involves a transcriptional cascade with PPARγ being most important in SAT but less so in VAT, with increased angiogenesis also critical. The transcription factor, Islet1, is fairly specific to VAT and *in vitro* inhibits adipocyte differentiation. The physiological importance of Islet1 requires further study. Basic control of differentiation is similar in BAT but important differences include the effect of PGC-1α on mitochondrial biosynthesis and upregulation of UCP1; also PRDM16 plays a pivotal role in expression of the BAT phenotype. Modulation of the capacity or function of these different adipose tissue depots, by altering adipocyte differentiation or other means, holds promise for interventions that can be helpful in human disease, particularly cardiometabolic disorders associated with the world wide explosion of obesity.

## Introduction

Subcutaneous adipose tissue accounts for about 85% of all body fat in people of a wide range of adiposity (1). Different human fat depots play contrasting physiological and pathophysiological metabolic roles with brown adipose tissue (BAT) being beneficial, subcutaneous white adipose tissue (SAT) potentially favorable, and visceral white adipose tissue (VAT), together with “ectopic” tissue lipid, potentially harmful (2). Variation in these different lipid depots may therefore impact significantly on metabolic health. Defining the nature of these variations may improve our understanding of and, hopefully in the future, lead to enhanced therapy or prevention of cardiometabolic disease. Here, we focus particularly on the role of adipocyte differentiation in this regard.

## Transcriptional Regulation of Adipocyte Differentiation in Different Fat Depots

### Characteristics of white adipose tissue and white adipogenesis

#### White adipose tissue

The primary metabolic role of white adipose tissue (WAT) is to store nutrients in the form of triglycerides so that it can be released during times of energy demand such as starvation or exercise. Adipose tissue is also recognized as an important endocrine organ and produces and secretes a number of peptides and other factors, which are known as adipokines. WAT is composed primarily of tightly packed, large spherical adipocytes (also called unilocular fat cells as opposed to multilocular adipocytes present in BAT), supported by a richly vascularized loose connective tissue. Adipogenesis, the differentiation of fibroblast-like mesenchymal stem cells (MSCs) into adipocytes, plays a central role in regulation of whole body energy metabolism. At the cellular level, adipogenesis is generally described as a two-step process: a commitment step, wherein committed adipocyte progenitors (or preadipocytes) are generated from multipotent MSCs, and a differentiation step, wherein pre-adipocytes acquire the features of mature, functional adipocytes (3).

#### Recruitment of new fat progenitor cells

Expansion and renewal of the white adipocyte pool in WAT is believed to rely on proliferation and self-renewal of mesenchymal precursor cells (4) that we term white adipocyte progenitors (WAPs). WAPs reside within the population of adipose stromal cells (ASCs) (5). Several studies have demonstrated that perivascular cells isolated from adipose tissue may harbor white fat progenitors (6–8). A study using PPARγ as a preadipocyte marker found that WAPs reside in the mural cell compartment of the adipose vasculature (9). Similarly, genetic labeling of a transcription factor that is enriched in fat cell precursors Zfp423 demonstrates a perivascular origin of pre-adipocytes, and this transcription factor is both necessary and sufficient for the development of a common precursor of white and brown adipocytes (10, 11). VE-cadherin (vascular endothelial) promoter-driven lineage-tracing experiments provide an independent line of evidence that both pericytes and murine endothelial cells can differentiate into pre-adipocytes and adipocytes (12).

#### Commitment

More light has been shed on adipocyte commitment studies in recent years. Several important transcription factors have been identified as regulators of preadipocyte determination, including Zfp423, Tcf7l1, and Ebf1 as positive transcription factors; and Zfp521, Wisp2 as negative transcription factors. Zfp423 induces adipose lineage commitment by amplifying the effects of the bone morphogenetic proteins (BMPs) signaling pathway, which is required for adipocyte lineage commitment (10, 13). Ebf1, a Zfp423 interactor (14, 15), is required for the generation of adipocyte progenitors (16, 17). However, the role of the interaction between Ebf1 and Zfp423 in preadipocyte commitment remains unknown. Zfp521 is a factor related to Zfp423, which represses preadipocyte commitment at least in part through direct inhibition of Ebf1 and subsequent repression of Zfp423 expression (17). Wisp2 binds directly to Zfp423 in the cytosol to inhibit its activity and negatively regulates preadipocyte commitment in mesenchymal precursor cells (18). In addition, extracellular WNT/WISP2 also regulates differentiation by preventing PPARγ activation via an unknown mechanism. Tcf7l1 acts in a different manner by responding to the confluence of the cells, mediating changes in structural proteins that regulate differentiation and in turn influence adipose commitment (19). What is interesting is that PPARγ activation is involved in mediating the effect of all currently identified commitment factors, which suggests that PPARγ is essential for preadipocyte determination in addition to its well known role as a “master regulator” of terminal adipocyte differentiation (20).

#### Differentiation

The transcriptional cascade that promotes differentiation has been well studied. Terminal differentiation is controlled by a tightly regulated transcriptional cascade where the transcription factors activate or repress the expression of each other in a sequential manner or by positive or negative feedback loops. Key players in this transcriptional cascade include CCAAT/enhancer-binding protein (C/EBP) family members (i.e., C/EBPα, C/EBPβ, and C/EBPδ), KLFs, CREB, Krox20, and PPARγ (20–22). C/EBPβ and δ act early in the terminal differentiation to induce the expression of PPARγ and C/EBPα. Differentiation is “locked in” by a positive feedback loop between PPARγ and C/EBPα (23, 24); a second positive feedback loop between PPARγ and C/EBPβ reinforces the decision toward differentiation (25).

### Characteristics of brown adipose tissue and brown adipogenesis

#### Brown adipose tissue

Recent studies have revisited the role of BAT in adults (26). Unlike WAT, BAT expresses the mitochondrial protein uncoupling protein 1 (UCP1) that enables dissociation of cellular respiration from ATP utilization, resulting in the release of stored electrochemical energy as heat, thereby fulfilling its role as a thermogenic organ. Contrary to the traditional view that BAT and WAT share a common developmental origin, recent studies revealed several unexpected developmental lineages giving rise to BAT and “brown fat-like” cells, orchestrated by novel transcriptional regulators.

#### PGC-1α and PRDM16

Brown adipocyte differentiation begins with commitment to adipogenesis by a cascade of transcriptional factor interactions similar to white adipocytes (see Characteristics of White Adipose Tissue and White Adipogenesis). However, PPARγ coactivator-1alpha (PGC-1α), the master regulator of mitochondrial biogenesis, is crucial in brown adipogenesis. PGC-1α drives the synthesis of UCP1 by stimulating its promoter (27). Recent studies have identified an array of nuclear receptor co-regulators that also stimulate brown adipogenesis, including *SRC-1*, *TIF2*, and *Twist-1*, which have been reviewed in detail (28).

A pivotal regulator of brown adipogenesis is PRD1-BF-1-RIZ1 homologous domain containing protein-16 (PRDM16). Ectopic expression of PRDM16 in fibroblasts, myoblasts, or pre-adipocytes is sufficient to induce the full brown fat transcriptome, including *PPARγ*, *UCP1*, and *PGC-1α* (29), with the emergence of a brown fat phenotype and function, including mitochondrial biogenesis and respiratory uncoupling. In contrast, depletion of PRDM16 in brown fat cells causes a near total loss of the brown characteristics (30).

#### Developmental origins

Ablation of PRDM16 increases the expression of MyoD, myogenin, myosin light chain, and muscle creatine kinase, together with morphological transformation of pre-adipocytes into myoblasts (31). These results are concordant with the identification of *Myf5*, a gene previously assumed to be expressed almost exclusively in committed skeletal muscle precursors, in interscapular BAT (29). Muscle-specific microRNAs are also expressed in brown but not in white adipocytes (32). Collectively, these results have underscored distinct origins of BAT and WAT, and consolidated the currently accepted view that classic brown adipocytes are more closely related to skeletal muscle than white adipocytes.

In addition to skeletal muscle, increasing evidence suggests that brown adipocytes may also be derived from other sources. Upon cold exposure, brown fat-like cells emerge within WAT in rodents (33, 34). These cells express UCP1 at levels indistinguishable from classic brown adipocytes. These brown fat-like cells within WAT are referred to as “brite” (brown-in-white) (33) or beige adipocytes (35). The developmental origin of brite/beige cells is a subject of ongoing debate. Based on the absence of an increase in cell number upon cold acclimatization, some argue that brite/beige cells arise from direct transformation or transdifferentiation from white adipocytes (36). Conversely, other studies support the presence of distinct inducible brite/beige precursors within WAT, which may also be *Myf5* positive (37).

#### Markers of different fat depots

A panel of molecular markers has been identified that delineate brown, white, and brite/beige adipocytes: *Lhx8* and *Zic1* for brown, *Tbx15* for brown/brite, *Hoxc9* and *Shox2* for brite/beige, *Hoxc8*, *Inhbb*, and *Dpt* for brite/white, and *Tcf21* for white adipocytes (38). Very recently, surface markers of each adipocyte type have been discovered; ASC-1, PAT2, and P2RX5, for white, beige/brite, and brown adipocytes, respectively (39).

In summary, brown adipocytes display a stronger myogenic signature than beige/brite adipocytes, while myogenic-related genes are absent in white adipocytes, implying a non-myogenic origin of brite adipocytes. This paradigm continues to evolve and is an area of intense research, with new evidence pointing to yet more diverse origins, such as brown adipocytes arising from hematopoietic stem cells within bone marrow (40) and brite/beige adipocytes differentiating from smooth muscle-like cells surrounding blood vessels (41).

## Regulation of Adipose Tissue Expansion in Different Fat Depots

### Genetic influence

There is a strong genetic contribution to overall adiposity (42), which is largely determined by SAT, so there are likely to be genes, which have a strong influence on adipose tissue differentiation/expansion as well as appetite regulation, though human monogenic forms of obesity of which the MC4R mutations are the commonest, appear mainly related to appetite and energy balance (2, 43). PPARγ mutations are an exception. The importance of PPARγ in regulating SAT is demonstrated by the effect of PPARγ agonists in causing expansion of the subcutaneous, but not visceral, fat compartment (44), and rare loss-of-function PPARγ mutations cause reduced subcutaneous (particularly gluteal) fat (45). VAT adipocytes express the PPARγ receptor in reasonable abundance but PPARγ is less able to promote adipocyte differentiation *in vitro* in adipose tissue from this compartment (46).

In fact, some ethnic groups, including Southern Indians and Australian aboriginals, seem to have a reduced ability to expand their peripheral subcutaneous fat in the face of energy surplus (47–50). The genetic control of this expansion, or lack thereof, is poorly understood although Lamin A must have a role as mutations in some regions of this gene cause reduced adipocyte differentiation *in vitro* and congenital lipodystrophy (51).

Again there is evidence from twin and other studies for strong genetic control over central abdominal (including visceral) fat (52, 53) independent of overall adiposity but without a clear understanding of the molecular mechanisms. We have reported the presence of the transcription factor Islet1 (important for development of islets, cardiac tissue, and neurons) in the stromovascular (preadipocyte-containing) fraction of VAT but not SAT and its expression is correlated in animals and humans with leanness (54). We have also recently demonstrated the ability of Islet1 to inhibit 3T3-L1 preadipocyte differentiation *in vitro* at least in part via downregulation of bone morphogenic protein 4 (BMP4) (55). Further work will be needed to determine whether Islet1 is a significant regulator of visceral adiposity in humans.

In contrast to the well-defined interscapular location of BAT in rodents, human BAT/beige fat is located in a fascial plane extending from the cervical to the supraclavicular/axillary regions, with smaller depots around the mediastinum, as well as pericardial, paravertebral, and suprarenal regions (56). In other words, both SAT (i.e., supraclavicular BAT) and VAT (i.e., suprarenal BAT) could harbor adipocytes with BAT-like features. Interestingly, adipose-specific ablation of PRDM16 inhibits formation of beige adipocytes in SAT of cold-exposed rodents (57). These animals rapidly gained SAT upon high fat feeding yet the excess “SAT” displayed a gene signature typical of VAT, characterized by inflammation and macrophage infiltration. These findings suggest complex interplay between adipose composition and location, and brown/beige/white adipogenesis may regulate adipose function in depot-specific manner.

### Hormonal influence

Hormonal influences (2) are important as indicated by the effect of estrogen increasing SAT (especially gluteal). Cortisol in excess increases central fat (both SAT and VAT) and growth hormone reduces subcutaneous adiposity. Again the mechanisms for these effects are not fully understood although increased lipolysis may be the predominant effect of growth hormone (58).

Estrogen not only favors expansion of SAT but also limits VAT (59) and the effect is therefore lost post-menopause (59). To what extent this action is related to modulation of adipocyte differentiation in the VAT compartment is unclear.

Numerous hormones modulate BAT activity. For example, thyroid hormone (60) and estradiol (61) stimulate mitochondrial biogenesis and brown adipogenesis, while testosterone (62) and cortisol (63) inhibit BAT proliferation and differentiation. Intriguingly, eosinophils through atypical macrophages also stimulate brite/beige adipogenesis in rodents (64). Recently, novel cytokines including irisin (65), fibroblast growth factor 21 (FGF21) (66), BMPs (67), and Meterorin-like (68) have been discovered, which independently regulate BAT and/or beige/brite fat function beyond classic SNS and/or pituitary–thyroid–adrenal axes, thereby opening new directions in BAT-based therapeutics (see Brown Adipose Tissue).

### Environmental influence

Exercise training (particularly aerobic) also reduces VAT and hepatic steatosis (69) to a greater extent than SAT (70, 71), presumably related to the greater lipolytic response of VAT adipocytes and simple depletion of triglyceride; however, an effect on limiting adipocyte differentiation/expansion is an additional possibility.

Studies have confirmed the presence of BAT and brown fat-like cells in humans (56) (see Brown Adipose Tissue). Whole body BAT function can therefore be expanded either through recruitment of classic BAT and/or brite/beige fat induction (i.e., fat browning). Cold exposure is the best-established mechanism for expanding BAT and beige depots: acute cold exposure elevates *UCP1* mRNA expression in both interscapular BAT and beige/brite fat within WAT, while prolonged cold exposure induces BAT hypertrophy and hyperplasia, and induces beige/brite adipogenesis (72). Although classically viewed as the mediator of cold exposure, the sympathetic nervous system (SNS) may not be the sole driver of cold-induced BAT recruitment.

Pharmacological studies have demonstrated remarkable proliferation/expansion of interscapular BAT during chronic beta-agonist exposure (73), and beige/brite adipogenesis can be induced within WAT following β3-agonist treatment, similar to results observed in cold exposure studies, thus supporting the role of SNS as a cold-mediator. However, cold may have a direct stimulatory effect on BAT, independent of SNS. Cold exposure, but not non-selective β-agonists in therapeutic dosage, activates BAT in humans, suggesting probable presence of non-SNS BAT-activating factors exerting effects during cold exposure. UCP1 expression is also increased in adipocytes exposed to cold temperature *in vitro* (74). The cell-autonomous response to cold further supports the presence of direct cold-mediated BAT expansion and/or fat-browning mechanisms.

## Implications for Pathophysiology/Therapy

Studies by our own and other groups have shown that abdominal obesity is associated with several negative effects including insulin resistance, cardiovascular disease, inflammation, and various cancers (75–78), which indicates that expansion of visceral fat is adverse, and conversely reduction of visceral fat mass should be beneficial.

Several factors may contribute to the adverse effects of VAT including a hyperactive secretome, inflammation, lipolysis, and transmission of fatty acids via the portal vein to the liver. Adipose tissue is an endocrine organ that secretes numerous proteins (adipokines) and lipids that have potent metabolic effects on other organs including muscle, liver, and brain (79, 80). The differential secretion of adipokines may account for the differing metabolic consequences of visceral vs. subcutaneous adiposity. Compared with SAT, VAT secretes lower amounts of metabolically beneficial adipokines including leptin and adiponectin, but higher amounts of detrimental or proinflammatory adipokines such as RBP4, TNF-α, MCP-1, IL-8, and IL-6 (81–83). Although adiponectin is expressed and secreted mainly by subcutaneous adipose tissue, lower adiponectin concentrations are related to visceral fat accumulation (84, 85). CRP levels are significantly related to waist circumference (WC), while MCP-1 is more highly associated with VAT compared with SAT (86, 87). Therefore, abdominal obesity is associated with reduced levels of adiponectin, and increased levels of inflammatory adipokines. MCP-1 can induce macrophage infiltration and activation in adipose tissue and these adipose tissue macrophages are postulated to be a major contributor to obesity-associated chronic low grade inflammation, which may contribute to the pathogenesis of obesity-induced insulin resistance (88, 89). Although insulin resistance has also been shown to occur independently of changes in AT inflammation (90), in which case it is possible that AT macrophage infiltration is serving an alternate function such as the clearance of dead adipocytes (91), which is an initial remodeling event required for AT regeneration and expansion in response to energy surfeit (92).

Lipolytic regulation also differs between VAT and SAT in that visceral adipocytes are more metabolically active; they have a greater lipolytic capacity (93–95) and, compared with subcutaneous abdominal or femoral adipose cells, they display greater catecholamine induced lipolysis and reduced suppression of lipolysis in response to both insulin and α2-adrenergic agonists (96, 97). The increased lipolytic capacity of VAT causes increased release of FFAs from VAT, potentially increasing hepatic gluconeogenesis (98) and contributing to ectopic lipid deposition in a range of tissues, contributing to insulin resistance (99). Fat accumulation in the liver (100) or muscle (101) is tightly associated with insulin resistance and type 2 diabetes. However, there is evidence that visceral fat is not as important as subcutaneous fat in supplying FFAs to the liver in lean or in most obese persons (102, 103). Additional studies are needed to definitively determine the relationship between individual abdominal fat depots and insulin resistance.

### Subcutaneous white adipose tissue

Although obesity, with increased SAT, is metabolically harmful, a lack of adipose tissue, especially SAT, can be as bad or worse. Various congenital or acquired lipodystrophies are characterized by hepatic steatosis, dyslipidemia, and insulin resistance with increased risk of diabetes (2, 104). This appears attributable to a combination of diversion of lipid away from an inadequate adipose tissue reservoir to “ectopic sites” such as liver and muscle, and also to a lack of secretion of the “favorable” adipokines leptin and adiponectin; the low leptin levels explain increased appetite and inappropriately positive energy balance and reduced levels of both adipokines contribute to reduced activity of AMP-activated protein kinase (AMPK), which promotes lipid oxidation (2). Human lipodystrophies used to be rare but HIV lipodystrophy, strongly associated with the earlier HIV antiviral drugs, has been common in subjects treated for HIV (105); again the molecular mechanisms involved are not clearly understood but may involve disturbances of adipocyte PPARγ and GLUT4 activity.

Conversely, PPARγ agonists such as pio- and rosiglitazone expand SAT, which appears to “sequester” excess lipid and lower circulating non-esterified fatty acids (NEFA) levels. The resulting metabolic improvement is also contributed to by increased adiponectin secretion; the net result being improved insulin sensitivity and prevention/improvement of type 2 diabetes (106). Of course the weight gain has negative musculoskeletal and cosmetic implications and the enhanced adipocyte differentiation may be at the expense of reduced osteoblast differentiation in bone with consequent reduction of bone density (107).

In treating ordinary obesity, energy restriction is paramount but increased exercise assists loss of adipose tissue rather than lean mass (2). The importance of energy restriction rather than simple reduction of SAT mass was clearly demonstrated by the complete lack of metabolic benefit from removal of ~10 kg of subcutaneous fat in obese subjects by liposuction (108). Thus, simple depletion of the SAT “reservoir” is of no value and the benefit of energy restriction by diet, bariatric surgery, or other means would seem to be dependent on reduction of hepatic, intramyocellular, and visceral lipid. In regard to the latter, surgical VAT resection in obese insulin resistant rats generates significant metabolic improvement (109) but evidence in humans is conflicting (2, 110).

So where do we go in using our knowledge of adipocyte differentiation to extend therapy beyond energy restriction? As indicated above, measures to reduce the SAT depot may do more harm than good; however, some animal models with reduced SAT do have a favorable phenotype, e.g., the c-Cbl deletion, where there is increased energy expenditure (111) and some genetic manipulations inhibiting adipose angiogenesis may improve insulin sensitivity/glucose tolerance (112) but some have the reverse effect (113) for reasons that are not clear but may include effects on appetite. Thus, with an increased knowledge of the molecular metabolism of these models – especially feedback effects on orexigenic pathways – it may be possible to design pharmacotherapy to reduce overall or subcutaneous fat, gain a favorable metabolic outcome, and get the musculoskeletal benefit of reduced weight.

### Visceral white adipose tissue

It is unclear if pharmacotherapy to specifically reduce VAT would be possible but VAT has a number of developmental genes, as well as Islet1, which are different from those in SAT (114, 115) so in theory such agents could be developed. The metabolic response would be uncertain but interesting and informative.

### Brown adipose tissue

One exciting prospect in this area is to increase the amount and/or activity of brown or “beige” fat. Animals with high BAT and/or beige/brite abundance are protected against obesity, diabetes, hepatic steatosis, and hyperlipidemia. BAT and/or beige/brite fat expanding therapeutic strategies are attractive in the combat against obesity and related disorders in humans.

In this regard, cold acclimation is an effective method of BAT recruitment in humans. Mild cold exposure at 16–19°C for 2–6 weeks increases BAT volume and activity in adults (116–118), augmenting cold-induced thermogenesis (116, 117), and resulting in reduced adiposity (117) as well as enhancement of post-prandial insulin sensitivity (118). Unfortunately, the natural tendency for thermal comfort could limit the applicability of cold exposure as a BAT expanding strategy.

Pharmacological BAT activation is an attractive alternative. The disappointing results of β3-agonist studies a decade ago on weight loss (119) could stem from the relatively low expression of β3-receptors in human BAT. The use of non-selective β-agonists for BAT expanding purposes is limited by inadvertent cross-stimulation of cardiac β-receptors. Non-SNS therapeutics based on newly discovered fat browning and/or BAT-activating cytokines have strong potential (56). FGF21 and irisin are particularly relevant as they are potent endocrine human BAT activators that are stimulated by cold exposure in adults (120). Whether FGF21 and irisin can be transformed into injectable recombinant proteins for obesity and/or diabetes treatment is under active research at present. It is important to point out that in all of these studies it is not possible to exclude non-cell autonomous effects for all of these perturbations including a central effect to repress food intake.

## Concluding Remarks

In summary, fat distribution (e.g., VAT vs. SAT) and composition (e.g., WAT vs. BAT) are both metabolic health determinants with therapeutic implications. Although adipogenesis dictates mature adipocyte phenotypes, adipogenesis at the tissue level defines fat distribution, and ultimately modulates organ function. Pinpointing crossroads in adipogenic checkpoints governing SAT/VAT expansion and WAT/BAT differentiation may ultimately open novel avenues in obesity treatment by sculpting a metabolically favorable whole body adipose distributional/compositional phenotype.

## Conflict of Interest Statement

The authors declare that the research was conducted in the absence of any commercial or financial relationships that could be construed as a potential conflict of interest.

## Abbreviations

ASC-1, Asc-type amino acid transporter 1; AT, adipose tissue; BAT, brown adipose tissue; BMPs, bone morphogenetic proteins; CRP, C-reactive protein, pentraxin-related; Dpt, dermatopontin; Ebf1, early B-cell factor 1; FGF21, fibroblast growth factor 21; Hoxc8, homeobox C8; Hoxc9, homeobox C9; IL-6, interleukin 6; IL-8, interleukin 8; Inhbb, inhibin, beta B; Lhx8, LIM homeobox 8; MCP-1, chemokine (C–C motif) ligand 2; MSCs, mesenchymal stem cells; Myf5, myogenic factor 5; MyoD, myogenic differentiation 1; NEFA, non-esterified fatty acids; P2RX5, purinergic receptor P2X, ligand-gated ion channel, 5; PAT2, solute carrier family 36 (proton/amino acid symporter), member 2; PGC-1α, PPARγ coactivator-1α; PRDM16, PRD1-BF-1-RIZ1 homologous domain containing protein-16; RBP4, retinol binding protein 4, plasma; SAT, subcutaneous white adipose tissue; Shox2, short stature homeobox 2; SNS, sympathetic nervous system; SRC-1, v-src avian sarcoma (Schmidt-Ruppin A-2) viral oncogene homolog; Tbx15, T-box 15; Tcf2, HNF1 homeobox B; Tcf7l1, transcription factor 7-like 1; TIF2, nuclear receptor coactivator 2; TNF-α, tumor necrosis factor; Twist-1, twist family bHLH transcription factor 1; UCP1, uncoupling protein 1; VAT, visceral white adipose tissue; WAT, white adipose tissue; Wisp2, WNT1 inducible signaling pathway protein 2; Zfp423, zinc finger protein 423; Zfp521, zinc finger protein 521; Zic1, Zic family member 1.
